# Differences in the global exposure, mortality and disability of low bone mineral density between men and women: the underestimated burden in men

**DOI:** 10.1186/s12889-023-15947-7

**Published:** 2023-05-29

**Authors:** Binxiang Zhu, Shian Hu, Jianfeng Guo, Zijian Dong, Yimin Dong, Feng Li

**Affiliations:** Department of Orthopedics, Tongji Hospital, Tongji Medical College, Huazhong University of Science and Technology, Jiefang Road, Qiaokou District, Wuhan, Hubei Province China

**Keywords:** Low bone mineral density, Osteoporosis, GBD 2019, Summary exposure value, Mortality, Disability-adjusted life year

## Abstract

Talking about osteoporosis, we tend to focus on post-menopause women who are at increased risk due to estrogen depletion, while less attention has been paid to the disease in men. Currently, there is a lack of understanding about the difference of osteoporosis incidence and burden by sex. In this study, we used data from the Global Burden of Disease Study 2019 (GBD 2019) to compare the difference in the prevalence and burden of low bone mineral density (LBMD) between men and women, by location, year, age and socio-demographic index. We found the prevalence of LBMD was higher in women than in men. However, the age standardized mortality rate was greatly higher in men than in women. Using disability-adjusted life year (DALY) to measure the burden, we also observed higher age standardized DALY rate in men. Using sociodemographic index (SDI) as the measure of social development level, we found that higher mortality and DALY rates were mainly seen in middle and high SDI countries. Falls were the leading cause for of deaths and disabilities in both men and women with LBMD, followed by transport injuries. Fall-related mortality was higher in women, while transport injuries caused more deaths and disabilities in men. Conclusively, more attention should be paid to osteoporosis in men, and related policies, clinical practices, and guidelines are in need to reduce the burden of LBMD and osteoporosis in men.

**Supplementary Information:**

The online version contains supplementary material available at 10.1186/s12889-023-15947-7.

## Introduction

Osteoporosis is an age-related endocrine disorders that predispose patients at increased risk of severe complications, such as fragile hip and vertebral fractures [1]. Osteoporosis and the related complications represent a huge threat to people’s well-being and import great burden to individual and social economic burden [2, 3]. For example, the cost of osteoporotic fractures reaches 17 billion dollars in the US every year, adding great burden to the healthcare systems [1].

It is well acknowledged that post-menopause women are at increased risk of osteoporosis, due to estrogen deficiency. Many clinical guidelines and studies have been focused on the screening and treatment of osteoporosis in women. In men, the related guidelines and clinical practice are relatively lacking. Specially, there is a lack of high-level evidence to guide when to screen for and treat osteoporosis in men. Indeed, osteoporosis in men may have been overlooked for too long [4]. A recently published study revealed that men have lower osteoporosis prevalence, but higher mortality and disability rates in men at the global level [3]. Another study suggested that the incidence of vertebral osteoporosis in men is two times lower than that in women, but is higher in middle-aged men [5]. Despite these reports, this is a lack of understanding about the sex differences in the prevalence and burden of osteoporosis by country, year, age, and SDI. A comprehensive comparison of the epidemiology and burden of osteoporosis and their trends in the past three decades between men and women at the global, regional and national level will provide valuable information for policy making and healthcare practice.

GBD 2019 provides a valuable source to estimate the epidemiology and burden of disease and injuries. In this study, we used data from GBD 2019 to explore the sex differences of osteoporosis. We reported the prevalence, mortality and DALY rates of osteoporosis for men and women at the global, regional, and national levels. We also compared the sex differences by age, year, and SDI. To the best of our knowledge, this is the first comprehensive study to compare the differences of osteoporosis prevalence and burden between men and women. We hope that this study could help to gain a better understanding of osteoporosis and provide valuable information for policy making and clinical practice.

## Methods

### Data source and access

The datasets generated and analyzed during the current study are available in the publicly accessible database Global Burden of Disease Study 2019 (GBD 2019). This study estimates the incidences, prevalence and burden of 369 diseases and injuries, and 84 risk factors by location, age, year, sex, and SDI [6]. GBD 2019 collected the raw data from civil registration, hospital records, household surveys, and vital statistics to estimate the prevalence and burden of LBMD. In this study, we explored the differences in the prevalence and burden of LBMD between men and women. Bone mineral density in GBD 2019 was measured at femoral neck by dual X-ray absorptiometry, in g/cm^2^. The data related to LBMD are standardized by age, which means that the demographic data were processed according to the same standard world age composition. This strategy helps to eliminate the influence of age structures of the population on the incidence and burden of specific disease and make it reasonable for trans-population comparisons. Other specific data of LBMD include summary exposure value (SEV), SDI, disability-adjusted life years (DALYs), and mortality rate by year, location, age, and sex.

### Summary exposure value

In the GBD study, SEV is an indicator of prevalence of LBMD. It is calculated by spatiotemporal Gaussian process regression (ST-GPR), which represents the weighted risk factor prevalence of LBMD [6]. SEV ranges from 0 to 100, with 0 indicating that the entire population have the lowest prevalence of LBMD, and 100 indicating the population have the highest prevalence. The increase in SEV indicates an increase in the prevalence rate of LBDM in a specific population, and vice versa.

### Socio-demographic index

SDI was developed by the GBD study to reflect the socio-demographic development level of a specific county or region (Results were shown in Supplementary Table 1). SDI takes into account per capita income, average education years, and fertility rate of women under 25 years old [6]. It represents the socio-development level of a country and indicates its effects on health outcomes. The SDI was divided into five levels in thus study: low SDI, low-middle SDI, middle SDI, high-middle SDI, and high SDI.

### The burden of LBMD

The burden of LBMD were measured by mortality and disability-adjusted life years (DALYs). DALYs refer to loss of life or reduction of ability life. DALYs is measured by years and consists of two parts. One is years of life lost (YLL), which means years of lost life due to premature death. Another one is years of lived with disability (YLD), which represents years of healthy life lost due to disability. DALYs are the sum of YLL and YLD. As DALY combines the influence of both disability and mortality to represent disease burden, this measure can represent the influence of disease on people's health more comprehensively and accurately, and provide a more reliable measurement of disease burden for the reference of policy making and clinical practice.

### Causes of LBMD-related disability and deaths

Patients with LBMD are more likely to experience fractures due to external causes, which leads to great disabilities and even death in such population. In GBD 2019, there external causes were classified into falls, road injuries, conflict and terrorism. Road injuries included pedestrian road injury, cyclist road injury, motorcyclist road injury, and motor vehicle road injury. In this study, we presented the ASMR and ASDR in patients with LBMD due to these causes for both men and women, and we compared the cause-related burden of LBMD between men and women.

### Statistical analysis

The age-standardized data of SEV, DALY and mortality rate were estimated by DisMod-MR 2.1, a Bayesian meta-regression tool. DisMod-MR 2.1 also produced the 95% uncertainty interval (95% UI) for each estimate. The trends of the age standardized DALY and mortality rates were represented by estimated annual percentage change (EAPC). EAPC was calculated based on a linear regression model, which was fitted as Y = α + βx + ε. In the model, the x-axis is the calendar year, while the y-axis indicates the ln (age standardized rates). EAPC was calculated as EAPC = 100*(exp(β) − 1). We also calculated the 95% confidence of each EAPC. An increasing trend was determined when the EAPC and the lower limit of the 95% CI were above zero, and vice versa for a decreasing trend. All the data processing and visualization were performed in the R software (version 4.1.2).

## Result

### The global and regional age-standardized SEV of LBMD between men and women

**Fig. 1:**
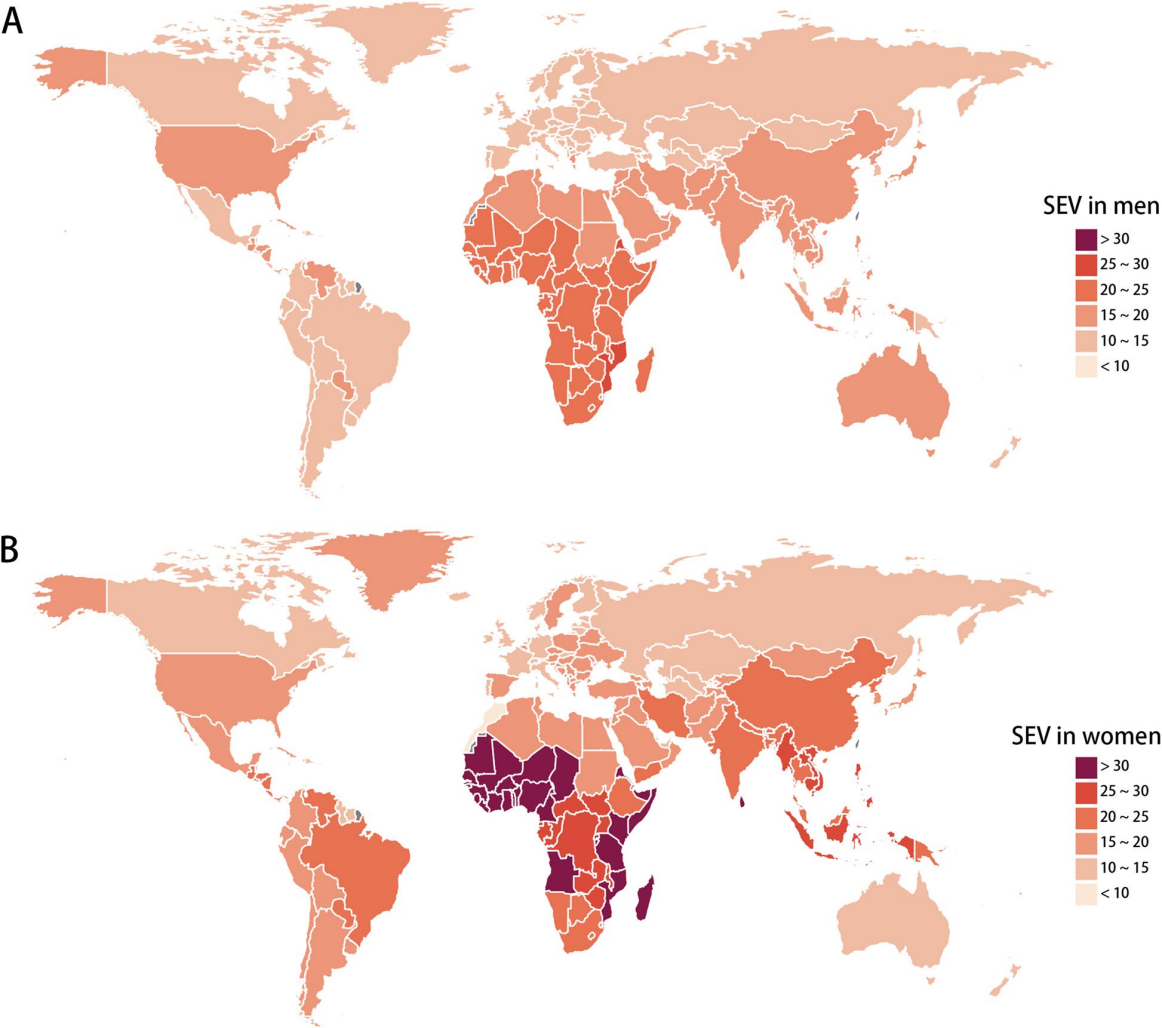
Global exposure to low bone mineral density. Age standardized SEV of LBMD, for men (**A**) and women (**B**) in 204 countries and territories in 2019. Abbreviations: SEV, summary exposure value

The SEV data were presented at the global level and for 22 regions (Table 1). Globally, the SEV of LBMD in 2019 in men was 11.3 (95% UI, 7 ~ 17.6), lower than that in women (20.7; 95% UI, 15 ~ 27.3). Higher SEV in women was also seen in Western Sub-Saharan Africa, Southeast Asia, Central Sub-Saharan Africa, Oceania, and Central Latin America. The highest SEV was seen in Western Sub-Saharan Africa for both men (16.8; 95% UI, 11.3 ~ 23.7) and women (32.8; 95% UI, 25.7 ~ 40.7). Eastern Europe had the lowest SEV for men (8.2; 95% UI, 4.6 ~ 13.6) and Western Europe had the lowest SEV for women (13.6; 95% UI, 8.9 ~ 19.8). From 1990 to 2019, the global SEV decreased for both men (EAPC, -0.34; 95% CI, -0.37 ~ -0.3) and women (EAPC, -0.11; 95% CI, -0.13 ~ -0.08). However, Oceania showed slightly increasing trends of SEV for men (EAPC, 0.01; 95% CI, -0.04 ~ 0.06), while women showed evident increasing trends in High-income North America (0.41; 95% CI, 0.26 ~ 0.57) (Supplementary Fig. 1). At the country level, high SEV in men and in women were mainly seen in countries located in Africa, Southeast, and Latin America (Fig. 1). Among the 204 countries, higher SEV in women was seen in all the of them, except for Morocco (Supplementary Table 2). Large differences in SEV between women and men were mainly seen in countries in Southeast Asia and West Africa (Supplementary Fig. 2).

**Table 1:** Global and regional age-standardized SEV of low bone mineral density in 2019 and the temporal trends from 1990 to 2019 for men and women

Location	Man		Woman	
	Age-standardized SEV per 100 in 2019 (95% UI)	EAPC from 1990 to 2019 (95% CI)	Age-standardized SEV per 100 in 2019 (95% UI)	EAPC from 1990 to 2019 (95% CI)
Global	11.3 (7 to 17.6)	-0.34 (-0.37 to -0.3)	20.7 (15 to 27.3)	-0.11 (-0.13 to -0.08)
Andean Latin America	9 (5 to 15.1)	-0.55 (-0.59 to -0.52)	16 (10.4 to 22.7)	-0.43 (-0.46 to -0.4)
Australasia	10.3 (5.6 to 16.5)	-0.54 (-0.61 to -0.48)	14 (8.9 to 20.7)	-0.44 (-0.46 to -0.42)
Caribbean	9.5 (5.2 to 15.5)	-0.48 (-0.51 to -0.45)	14.3 (9.3 to 20.4)	-0.43 (-0.46 to -0.4)
Central Asia	7.9 (4.2 to 13.1)	-0.23 (-0.27 to -0.18)	14.8 (9.7 to 21.4)	-0.15 (-0.18 to -0.11)
Central Europe	6.3 (3 to 11.1)	-0.43 (-0.48 to -0.38)	16.6 (11.3 to 23.2)	-0.31 (-0.34 to -0.29)
Central Latin America	8.9 (4.8 to 14.7)	-0.52 (-0.55 to -0.48)	19.8 (14 to 26.9)	-0.24 (-0.27 to -0.21)
Central Sub-Saharan Africa	17 (11.3 to 24.2)	-0.13 (-0.17 to -0.09)	28.5 (21.6 to 36.3)	-0.05 (-0.08 to -0.02)
East Asia	12.4 (7.7 to 19.2)	-0.74 (-0.84 to -0.64)	22.9 (17.1 to 29.6)	-0.55 (-0.61 to -0.49)
Eastern Europe	8.2 (4.6 to 13.6)	-0.03 (-0.08 to 0.01)	14.8 (9.9 to 20.9)	-0.18 (-0.21 to -0.15)
Eastern Sub-Saharan Africa	18.1 (12.5 to 25.3)	-0.29 (-0.31 to -0.26)	28.6 (21.8 to 36.2)	-0.16 (-0.18 to -0.13)
High-income Asia Pacific	10.3 (6.2 to 16.3)	-0.36 (-0.42 to -0.29)	18.7 (13.6 to 24.8)	-0.28 (-0.32 to -0.23)
High-income North America	11.2 (6.8 to 17.8)	-0.03 (-0.26 to 0.19)	17.8 (12.5 to 24.7)	0.41 (0.26 to 0.57)
North Africa and Middle East	11.9 (7.4 to 18.2)	-0.22 (-0.25 to -0.19)	18 (12.8 to 24.5)	-0.24 (-0.26 to -0.21)
Oceania	9.3 (5.1 to 15.3)	0.01 (-0.04 to 0.06)	20.2 (14.1 to 27.1)	-0.06 (-0.1 to -0.02)
South Asia	12.1 (7.6 to 18.4)	-0.36 (-0.39 to -0.32)	22 (16.1 to 28.8)	-0.24 (-0.3 to -0.18)
Southeast Asia	11.2 (6.7 to 17.6)	-0.32 (-0.35 to -0.29)	26 (19.9 to 32.8)	-0.13 (-0.16 to -0.11)
Southern Latin America	9.6 (5.4 to 15.7)	-0.39 (-0.43 to -0.35)	17.2 (11.8 to 24.1)	-0.45 (-0.48 to -0.43)
Southern Sub-Saharan Africa	15.8 (10.4 to 22.6)	-0.38 (-0.42 to -0.33)	22.9 (16.6 to 30.1)	-0.18 (-0.22 to -0.14)
Tropical Latin America	10 (5.7 to 16)	-0.57 (-0.61 to -0.53)	20.3 (14.1 to 27.6)	-0.35 (-0.38 to -0.32)
Western Europe	8.3 (4.5 to 14)	-0.32 (-0.37 to -0.27)	13.6 (8.9 to 19.8)	-0.28 (-0.31 to -0.25)
Western Sub-Saharan Africa	16.8 (11.3 to 23.7)	-0.19 (-0.22 to -0.16)	32.8 (25.7 to 40.7)	-0.16 (-0.17 to -0.14)

Data are presented in SEV with 95% UI. The SEV ranges from 0 to 100. SEV of 0 indicates that the total population is at minimum risk, while SEV of 100 indicates all the population is at maximum risk. For EAPC, data are presented in EAPC value with 95% confidence interval

*SEV* Summary exposure value, *EAPC* Estimated annual percentage change, *UI* Uncertainty interval, *CI* Confidence interval

### The global and regional age-standardized DALY of LBMD between men and women

**Fig. 2:**
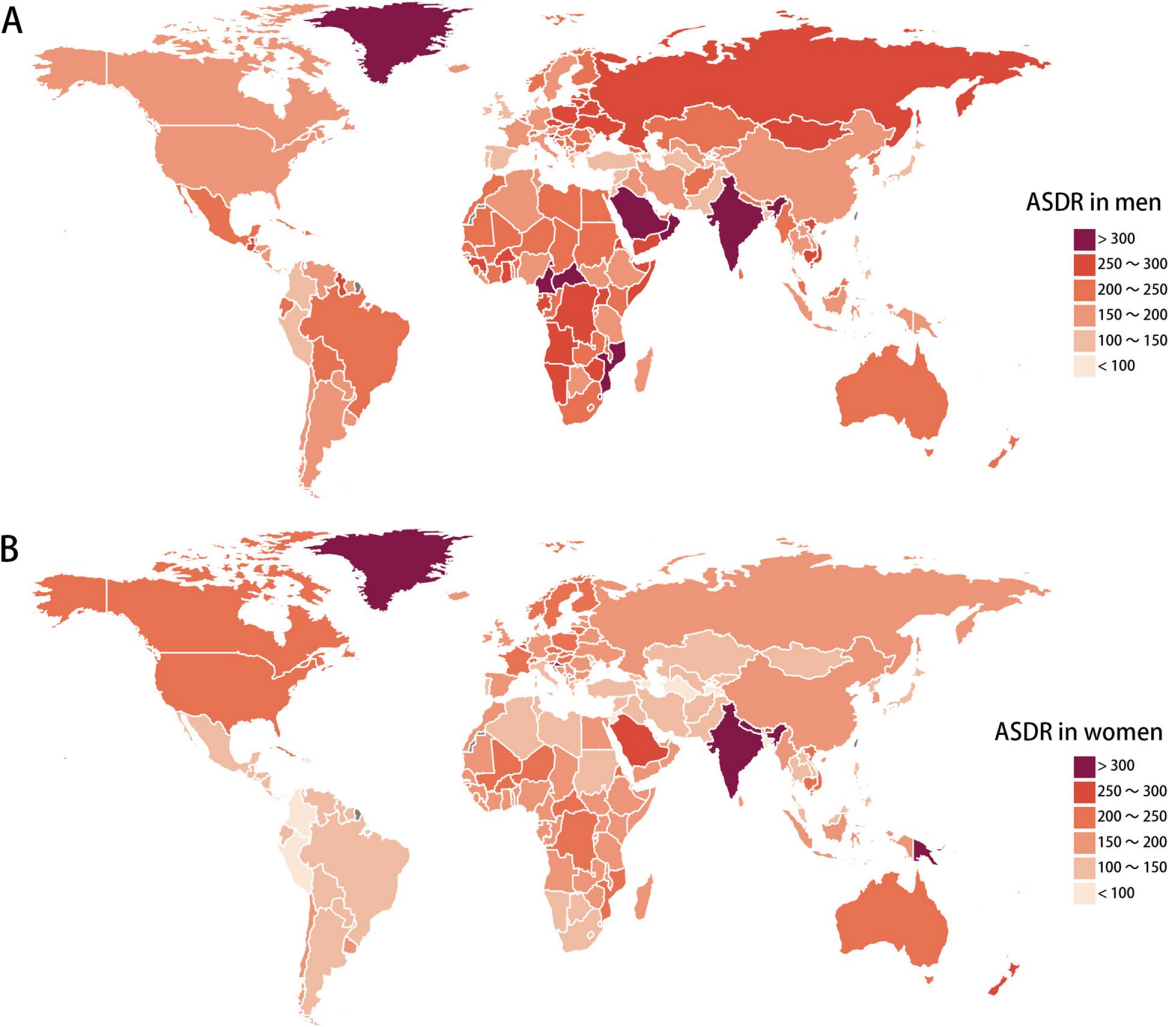
Global age standardized DALY rate of low bone mineral density. The all-cause ASDR per 100,000 associated with LBMD, for men (**A**) and women (**B**) in 204 countries and territories in 2019. Abbreviations: DALY, disease adjusted life year; ASDR, age standardized DALY rate

Table 2 and Fig. 2 show the distribution of DALY in the regional and national level. Globally, the ASDR of LBMD in 2019 was 212.7 (95% UI, 173.1 ~ 250.9) per 100,000 in men, higher than that in women (197.9 per 100,000; 95% UI, 158.5 ~ 242.0). At the regional, men demonstrated higher ASDR than women in Southern Sub-Saharan Africa, Eastern Europe, Tropical Latin America, while women had higher ASDR in in Oceania, South Asia, High-income North America, Western Europe and Australasia. The highest ASDR was seen in South Asia for both men (293.3 per 100,000; 95% UI, 236.2 ~ 350.1) and women (371.4 per 100,000; 95% UI, 298.7 ~ 444.9). High-income Asia Pacific had the lowest ASDR for men (139.6 per 100,000; 95% UI, 110.1 ~ 176.9) and Andean Latin America had the lowest ASDR for women (106.8 per 100,000; 95% UI, 84.2 ~ 128.1). From 1990 to 2019, the global ASDR decreased for both men (EAPC, -0.36; 95% CI, -0.42 ~ -0.31) and women (EAPC, -0.31; 95% CI, -0.37 ~ -0.26), but East Asia and Australasia showed evident increasing trends of ASDR for men, with an EAPC of 0.42 (95% CI, 0.27 ~ 0.56) and 0.26 (95% CI, 0.21 ~ 0.31), respectively. Women showed increasing trends in Australasia (EAPC, 0.55; 95% CI, 0.46 ~ 0.63), Oceania (EAPC, 0.53; 95% CI, 0.39 ~ 0.68), High-income North America (EAPC, 0.44; 95% CI, 0.31 ~ 0.57), and East Asia (EAPC, 0.1; 95% CI, 0 ~ 0.19) (Supplementary Fig. 3). In the country level, the highest ASDR in men was seen in Saudi Arabia and in women was seen in Solomon Islands. Men had higher ASDR than women in 78% of all the countries, and the largest difference was seen in Saudi Arabia, Lesotho, and Central African Republic (Supplementary Table 3). These results suggested men tend to have higher disability rate due to osteoporosis than women.

**Table 2:** Global and regional ASDR of low bone mineral density in 2019 and the temporal trends from 1990 to 2019 for men and women

Location	Man		Woman	
	ASDR per 100,000 in 2019 (95% UI)	EAPC from 1990 to 2019 (95% CI)	ASDR per 100,000 in 2019 (95% UI)	EAPC from 1990 to 2019 (95% CI)
Global	212.7 (173.1 to 250.9)	-0.36 (-0.42 to -0.31)	197.9 (158.5 to 242)	-0.31 (-0.37 to -0.26)
Andean Latin America	178.5 (139.8 to 216.1)	-0.54 (-0.64 to -0.45)	106.8 (84.2 to 128.1)	-0.22 (-0.28 to -0.16)
Australasia	223.7 (172.9 to 289.8)	0.26 (0.21 to 0.31)	244.4 (181.7 to 325.7)	0.55 (0.46 to 0.63)
Caribbean	190 (155.2 to 223.7)	-0.34 (-0.51 to -0.18)	149.5 (117.3 to 181.1)	-0.01 (-0.1 to 0.09)
Central Asia	169.8 (131.5 to 209.7)	-0.52 (-0.69 to -0.35)	112.5 (86.4 to 143.5)	0.04 (-0.11 to 0.19)
Central Europe	251.9 (190.4 to 319.8)	-1.18 (-1.22 to -1.15)	211.1 (159.5 to 275.3)	-1.41 (-1.55 to -1.27)
Central Latin America	191.2 (153.7 to 227.1)	-1.13 (-1.27 to -0.99)	122.9 (98.4 to 151.5)	-1.05 (-1.22 to -0.88)
Central Sub-Saharan Africa	278.1 (211.2 to 343)	-0.5 (-0.57 to -0.43)	195.2 (154.2 to 317)	-0.27 (-0.31 to -0.23)
East Asia	188 (144 to 228.3)	0.42 (0.27 to 0.56)	160.3 (124.8 to 198.1)	0.1 (0 to 0.19)
Eastern Europe	272.2 (208.1 to 350.9)	-1.09 (-1.41 to -0.76)	182.1 (136.5 to 239.4)	-0.81 (-1.03 to -0.58)
Eastern Sub-Saharan Africa	226.6 (193.1 to 261)	-0.69 (-0.74 to -0.64)	179.7 (151.7 to 209)	-0.47 (-0.54 to -0.4)
High-income Asia Pacific	139.6 (110.1 to 176.9)	-1.39 (-1.49 to -1.29)	139 (103.8 to 184.9)	-0.71 (-0.81 to -0.61)
High-income North America	188.7 (153.6 to 228)	0 (-0.04 to 0.03)	223.6 (172.8 to 286.8)	0.44 (0.31 to 0.57)
North Africa and Middle East	207.4 (155.6 to 245.1)	-0.84 (-0.88 to -0.81)	138.8 (110.4 to 167.4)	-0.33 (-0.37 to -0.28)
Oceania	159.6 (120.8 to 199.1)	-0.23 (-0.44 to -0.01)	299.9 (161.2 to 395.6)	0.53 (0.39 to 0.68)
South Asia	293.3 (236.2 to 350.1)	-0.09 (-0.22 to 0.04)	371.4 (298.7 to 444.9)	-0.33 (-0.45 to -0.21)
Southeast Asia	192.8 (157.5 to 223.7)	-0.72 (-0.76 to -0.68)	160 (124.6 to 190.1)	-1.19 (-1.29 to -1.1)
Southern Latin America	164.7 (131.5 to 202)	-0.63 (-0.73 to -0.54)	138.1 (106.2 to 176.7)	-0.45 (-0.53 to -0.36)
Southern Sub-Saharan Africa	220.2 (182.7 to 248.3)	-1.18 (-1.54 to -0.82)	115.5 (95.7 to 134.6)	-0.98 (-1.1 to -0.87)
Tropical Latin America	229.7 (188.9 to 265)	-0.66 (-0.75 to -0.58)	144.6 (117.5 to 173.6)	-0.39 (-0.55 to -0.24)
Western Europe	160.9 (127 to 201.4)	-0.89 (-0.95 to -0.84)	183.9 (137.6 to 238.7)	-0.43 (-0.52 to -0.34)
Western Sub-Saharan Africa	213.9 (175.1 to 257.7)	0.01 (-0.04 to 0.05)	168.6 (140.1 to 200.3)	-0.16 (-0.19 to -0.13)

Data are presented in ASDR with 95% UI. For EAPC, data are presented in EAPC value with 95% confidence interval

*DALY* Disease adjusted life year, *ASDR* Age standardized DALY rate, *EAPC* Estimated annual percentage change, *UI* Uncertainty interval, *CI* Confidence interval

### The global and regional age-standardized mortality rate (ASMR) of LBMD between men and women

**Fig. 3:**
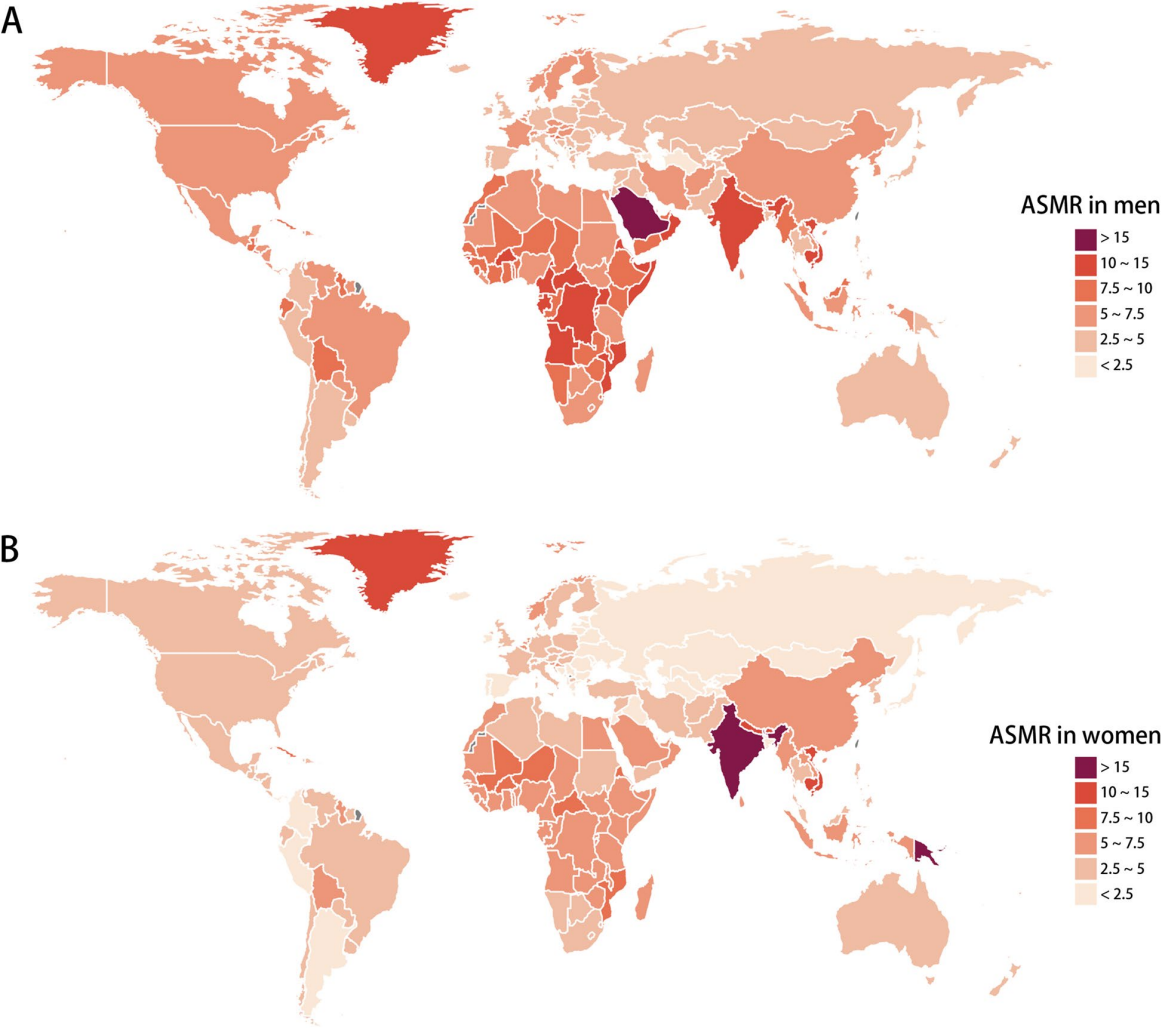
Global exposure to low bone mineral density. The all-cause ASMR per 100,000 associated with LBMD, for men (**A**) and women (**B**) in 204 countries and territories in 2019. Abbreviations: ASMR, age standardized mortality rate

The ASMR of countries and regions is shown in Table 3 and Fig. 3. In 2019, men also demonstrated higher ASMR than women globally (6.3 per 100,000 in men VS 5.2 per 100,000 in women). Regions with relatively higher ASMR for men were Sub-Saharan Africa, Andean Latin America, Central Latin America, Eastern Europe, and Central Asia. Central Sub-Saharan Africa had the highest ASMR of men (10.5 per 100,000; 95% UI, 7.9 ~ 12.8) and Oceania had the highest ASMR of women (13.2 per 100,000; 95% UI, 3.2 ~ 20.5). From 1990 to 2019, the global ASMR decreased for both men (EAPC, -0.19; 95% CI, -0.27 ~ -0.11) and women (EAPC, -0.21; 95% CI, -0.28 ~ -0.14). However, the ASMR in East Asia (EAPC in men, 0.98; 95% CI, 0.68 ~ 1.29; EAPC in women, 0.89; 95% CI, 0.47 ~ 1.31), High-income North America (EAPC in men, 0.88; 95% CI, 0.76 ~ 1; EAPC in women, 1.42; 95% CI, 1.23 ~ 1.62), and Australasia (EAPC in men, 0.87; 95% CI, 0.7 ~ 1.04; EAPC in women, 1.06; 95% CI, 0.88 ~ 1.25) showed striking growing trends for both sexes. In addition to the above areas, the ASMR in women also increased significantly in Oceania (EAPC, 0.39; 95% CI, 0.15 ~ 0.63), Central Asia (EAPC, 0.22; 95% CI, -0.07 ~ 0.5), and Tropical Latin America (EAPC, 0.08; 95% CI, -0.19 ~ 0.36) (Supplementary Fig. 4). Similar to ASDR, most countries (185 of 204) had higher ASMR in men than in women, except for India, Solomon Islands and Papua New Guinea (Supplementary Table 4). These results suggested men tend to have higher mortality due to osteoporosis than women.

**Table 3:** Global and regional ASMR of low bone mineral density in 2019 and the temporal trends from 1990 to 2019 for men and women

Location	Man		Woman	
	ASMR per 100,000 in 2019 (95% UI)	EAPC from 1990 to 2019 (95% CI)	ASMR per 100,000 in 2019 (95% UI)	EAPC from 1990 to 2019 (95% CI)
Global	6.3 (5.3 to 7.1)	-0.19 (-0.27 to -0.11)	5.2 (4 to 6.1)	-0.21 (-0.28 to -0.14)
Andean Latin America	5.3 (4.1 to 6.6)	-0.53 (-0.64 to -0.41)	3.1 (2.4 to 3.7)	-0.51 (-0.62 to -0.4)
Australasia	4.9 (4.1 to 5.5)	0.87 (0.7 to 1.04)	4 (3 to 4.8)	1.06 (0.88 to 1.25)
Caribbean	6.7 (5.5 to 7.8)	-0.29 (-0.47 to -0.1)	5.6 (4.2 to 6.8)	-0.24 (-0.42 to -0.06)
Central Asia	3.2 (2.6 to 3.6)	-0.26 (-0.53 to 0)	1.5 (1.2 to 1.7)	0.22 (-0.07 to 0.5)
Central Europe	4.7 (3.9 to 5.5)	-1.83 (-1.9 to -1.77)	3.2 (2.5 to 3.8)	-3.42 (-3.6 to -3.24)
Central Latin America	5.2 (4.2 to 6.2)	-1.81 (-1.96 to -1.65)	3.1 (2.5 to 3.6)	-2.11 (-2.35 to -1.87)
Central Sub-Saharan Africa	10.5 (7.9 to 12.8)	-0.52 (-0.59 to -0.44)	7.2 (5.5 to 12.4)	-0.22 (-0.27 to -0.17)
East Asia	6.4 (4.3 to 8.2)	0.98 (0.68 to 1.29)	5 (2.9 to 6.4)	0.89 (0.47 to 1.31)
Eastern Europe	3.8 (3 to 4.5)	-1.4 (-1.9 to -0.89)	1.8 (1.5 to 2.1)	-1.53 (-1.93 to -1.14)
Eastern Sub-Saharan Africa	8.8 (7.6 to 10.1)	-0.53 (-0.61 to -0.45)	6.6 (5.4 to 7.6)	-0.35 (-0.38 to -0.31)
High-income Asia Pacific	3 (2.5 to 3.4)	-1.52 (-1.67 to -1.37)	1.6 (1.2 to 1.9)	-2.1 (-2.22 to -1.98)
High-income North America	5.3 (4.6 to 5.8)	0.88 (0.76 to 1)	3.9 (3.2 to 4.4)	1.42 (1.23 to 1.62)
North Africa and Middle East	6.2 (4.3 to 7.4)	-0.88 (-0.94 to -0.82)	4.1 (2.9 to 4.9)	-0.29 (-0.47 to -0.11)
Oceania	4.9 (3.6 to 6.2)	-0.36 (-0.7 to -0.02)	13.2 (3.2 to 20.5)	0.39 (0.15 to 0.63)
South Asia	10.3 (8.2 to 12.4)	-0.41 (-0.58 to -0.23)	13.1 (9.8 to 16.2)	-0.7 (-0.94 to -0.46)
Southeast Asia	6.3 (4.8 to 7.4)	-0.67 (-0.72 to -0.62)	6.1 (3.8 to 7.4)	-1.36 (-1.47 to -1.25)
Southern Latin America	3.7 (3.2 to 4.1)	-0.91 (-0.96 to -0.87)	2.8 (2.3 to 3.2)	-0.7 (-0.88 to -0.52)
Southern Sub-Saharan Africa	6.6 (5.6 to 7.5)	-1.17 (-1.6 to -0.73)	3.3 (2.8 to 3.8)	-1.12 (-1.38 to -0.85)
Tropical Latin America	6.5 (5.5 to 7.2)	-0.46 (-0.65 to -0.26)	4.4 (3.4 to 5)	0.08 (-0.19 to 0.36)
Western Europe	4.5 (3.8 to 5)	-0.86 (-0.98 to -0.73)	3.6 (2.7 to 4.2)	-1.41 (-1.6 to -1.22)
Western Sub-Saharan Africa	8 (6.4 to 10.2)	-0.05 (-0.14 to 0.04)	6.5 (5.2 to 8.2)	-0.1 (-0.14 to -0.05)

Data are presented in ASMR with 95% UI. For EAPC, data are presented in EAPC value with 95% confidence interval

*ASMR* Age standardized mortality rate, *EAPC* Estimated annual percentage change, *UI* Uncertainty interval, *CI* Confidence interval

### The SEV, ASDR and ASMR of low bone mineral density in men and women by SDI

**Fig. 4:**
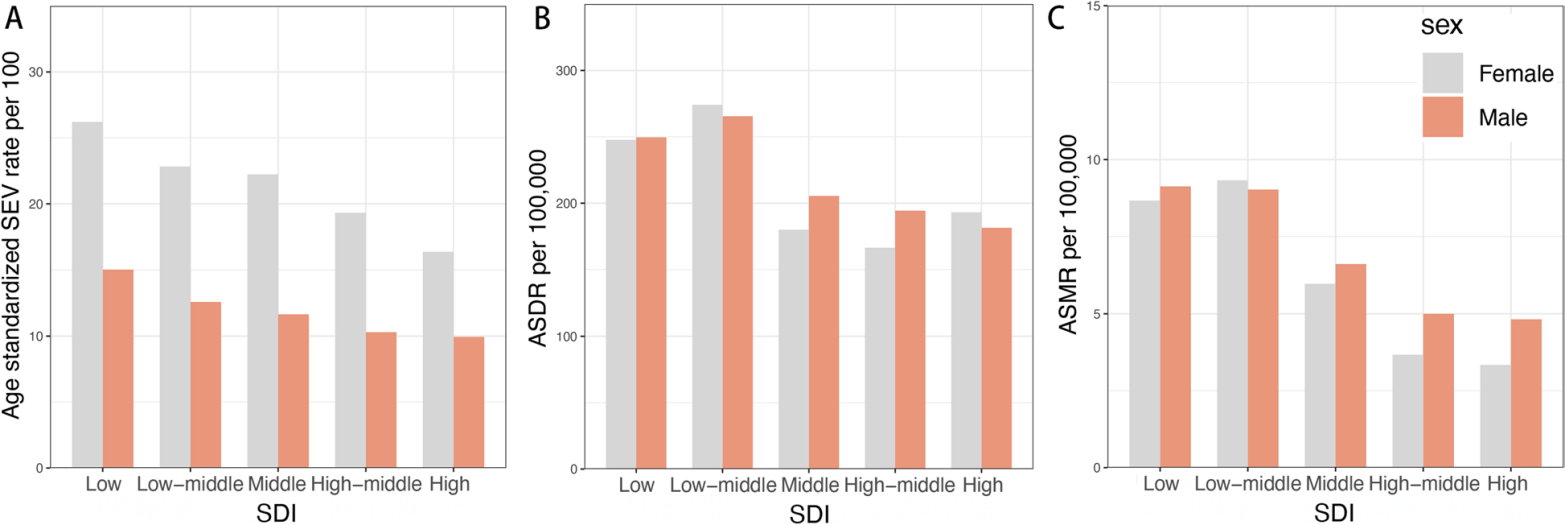
The exposure and burden of LBMD by SDI. The age standardized SEV, ASDR and ASMR of LBMD in different SDI regions in 2019. Results are showed for men and women worldwide. Abbreviations: SDI, socio-demographic index; SEV, summary exposure value; ASDR, age standardized DALY rate; DALY, disease adjusted life year; ASMR, age standardized mortality rate

Figure 4 showed the age-standardized SEV rate, ASDR, ASMR of LBMD by sex in different SDI regions. Women showed higher SEV than men in all SDI regions (Fig. 4A). Unlike SEV, ASMR and ASDR were comparable in low and low-middle SDI regions. However, middle and high-middle SDI regions showed higher ASDR and ASMR in men than in women. In addition to these two SDI regions, high SDI regions also showed higher ASMR in men. These results suggested that higher burden of LBMD in men are mainly seen in middle to high SDI regions.

### Differences in the causes of osteoporosis-related disability between men and women

**Fig. 5:**
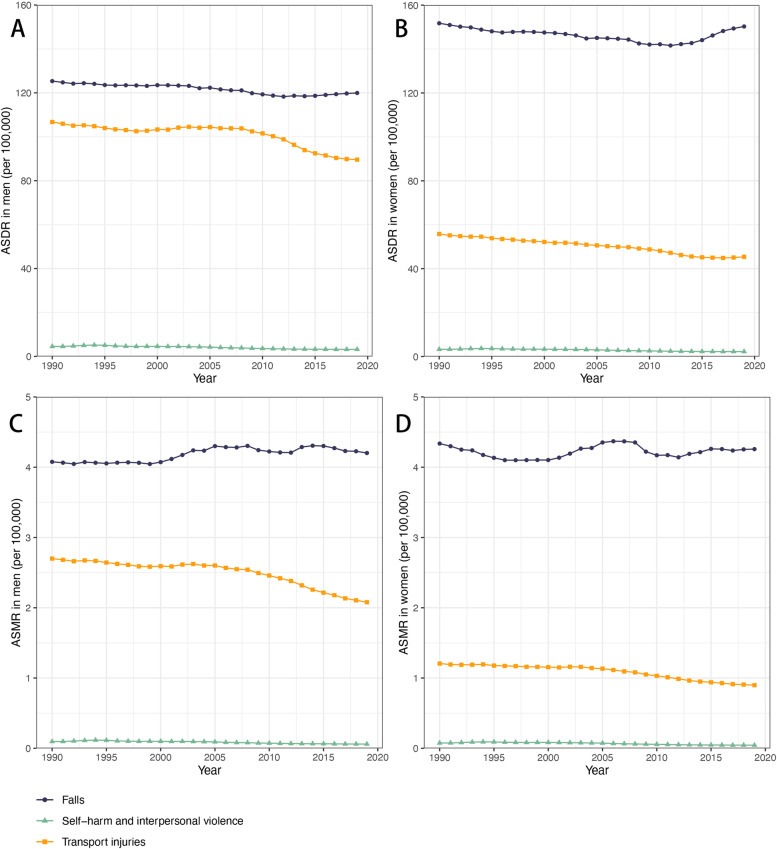
The causes of low bone mineral density disease burden. The three causes of ASDR and ASMR associated with low bone mineral density, for men (**A**, **C**) and women (**B**, **D**). Abbreviations: ASDR, age standardized DALY rate; DALY, disease adjusted life year; ASMR, age standardized mortality rate

In the GBD database, LBMD risk causes are divided into 3 categories: transport injuries, unintentional injuries and self-harm, and interpersonal violence. Transport injuries include several sub-categories: motor vehicle road injuries, cyclist road injuries, pedestrian road injuries, motorcyclist road injuries, other road injuries, and other transport injuries. Unintentional injuries are falls. In each year from 1990 to 2019, falls were the leading cause for ASMR and ASDR associated with LBMD for both men and women. In each year from 1990 to 2019, falls caused higher ASDR in women with LBMD than in men (Fig. 5A, B), while the ASMR associated with falls were similar between the two sexes (Fig. 5C, D). Transport injuries ranked as the second cause for disability and mortality for both men and women with LBMD, and transport injury related ASMR and ASDR were higher in men than that in women (Fig. 5 A-D).

### DALY and mortality attributable burden by age

**Fig. 6:**
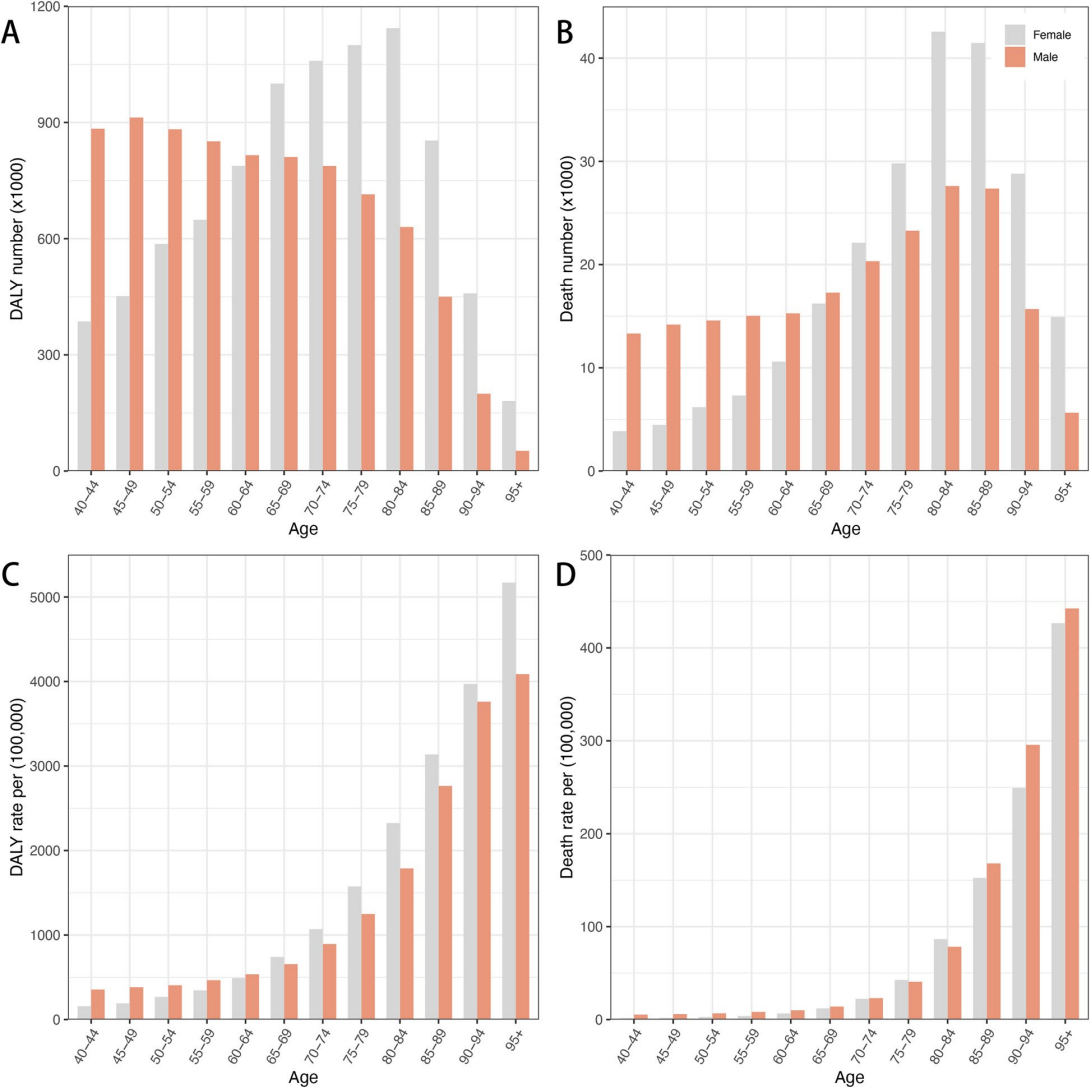
The exposure and burden of LBMD by age and sex. The disability number (**A**) and mortality number (**B**) in different age groups in 2019. The disability rate (**C**) and mortality rate (**D**) in different age groups in 2019. Abbreviations: DALY, disease adjusted life year

As LBMD mainly affects elderly people, the data regarding LBMD are available only for people aged 40 years or older in GBD 2019. With the increase of age, the absolute number of DALY decreased gradually in men, while in women, the DALY numbers increased with age until 85 years old (Fig. 6A). For LBMD related deaths, men and women showed similar changes in death numbers. Both sexes reached the maximum of death number at about 85 years of age, and then decreased (Fig. 6B). This may be related to the world population structure and the difference of living age between men and women. DALY and death rate due to osteoporosis increased greatly with age. Males had a higher DALY rate than females before 65 years of age, while the DALY rate of women exceeded that of men and increased sharply after 65 years of age (Fig. 6C). For mortality rate associated with LBMD, men showed a bit higher mortality rate than women for almost the whole lifespan (Fig. 6D).

## Discussion

In this study, we described the differences in the prevalence, DALY and mortality of LBMD between men and women in 2019 and the temporal trends from 1990 to 2019. Women showed higher SEV then men at the global level, as well as in most regions and countries, suggesting that osteoporosis is more likely to affect women than men. However, we found men demonstrated higher ASDR and ASMR than women, suggesting that osteoporosis related disease burden is heavier in men. Men and women also differed with each other in the causes of LBMD-related deaths and disabilities. Road injuries are more likely to be the cause of osteoporotic deaths and disabilities in men with LBMD, while fall-related osteoporotic deaths and disabilities were more common in women.

A few factors have been identified to increase the risk of developing LBMD or osteoporosis. Uncontrollable risk factors for osteoporosis include advanced age, female sex, menopause, family history, and race, while controllable risk factors include low body weight, low sex hormones, smoking, excessive drinking, lack of physical activity, dietary calcium and vitamin D deficiency [1, 7, 8]. Among these risk factors, female sex is associated with a twice higher risk of developing osteoporosis. This increased risk in women is explained by the lack of estrogen after menopause. Women aged over 70 years old have experienced long-term deficiency of estrogen, and overactivated activated osteoclasts and enhanced resorptive function are the main cause for bone loss in the absence of estrogen, which leads to osteoporosis [9]. Besides, the rate of hyperparathyroidism in postmenopausal women is also higher, which leads to hypercalcemia and bone diseases [10, 11]. A third reason is that women tend to live significantly longer than men, and the absolute number of osteoporosis in older women reached six times as many as that of men due to the higher proportion of women of advanced age [12].

However, after standardizing by age, we found the DALY and mortality rates were higher in men than that in women at the global level and in most regions and countries. Unlike women, LBMD-related absolute DALYs in men mainly came from those aged 65 years or younger, and the number of DALYs decreased with age. Accordingly, higher rate of DALY in men than in women was seen only in people younger than 65 years of age. Suggesting that LBMD-related disabilities in men mainly came from those in young people. One of the main reasons lies in the differences of work and physical activities between men and women. Although the BMD in men of young age is higher than in the old, it should also be noted that young men are more likely to participate in many high energy work, sports, or activities, which render them at increased risk of fracturs on a condition of decreased BMD with age. For the causes of osteoporotic fractures, age standardized mortality and DALY rate caused by transport injuries were both higher in men than that in women, which could be attributed to the fact that men are more likely to participate in vehicle- and road-related work or activities, including driving, cycling, and motorcycle riding. Additionally, higher mortality and DALY rates were seen in middle to high SDI countries, which may also be due to better road transportation facilities and more popularized vehicle use. Compared to people with normal BMD, people with decreased BMD are more likely to suffer fractures from accidental events during these work or activities, especially vertebral or hip fractures, which are usually associated with disabilities and increased mortality rate [13, 14].

The difference of hormonal effects on bone mass may also explain the difference of prevalence and burden of PBMD between men and women. In women, the loss of bone mineral density is accelerated by the loss of protective effects of estrogen on bone quality in the postmenopausal decade. Estrogen and testosterone have the same effect on bone mass in older men[9, 10, 15]. Androgens can maintain BMD by directly binding to androgen receptors or indirectly binding to estrogen receptors through aromatization to estrogen, and testosterone deficiency during aging is an important factor for bone loss in men [16]. The level of testosterone begin to drop by 1–2% annually since the age of 40 years [17], earlier than the age of menopause in women. Sex hormone binding globulin (SHBG) is a plasma glycoprotein that can reflect sex steroid levels. The serum testosterone concentration decreases with age, while the SHBG levels increase, which is related to many diseases, including osteoporosis [18]. A study of hormones and SHBG showed that high serum SHBG was significantly associated with increased risk of clinical spine fracture in elderly men (HR = 1.24; 95%CI, 1.12–1.37) [19]. Such differences are also reflected by the different changes of BMD with age between women and men. The Canadian Multicenter Osteoporosis Study suggested that women's lumbar spine BMD peaked at ages of 33 to 40 years, while men's lumbar spine BMD peaks at ages of 19 to 33 years, much earlier than women, suggesting that bone density in men begins to decrease earlier than women [20]. This earlier decrease renders men at increased risk of developing LBMD and osteoporosis during a wider range of the whole lifespan.

Men and women have different unhealthy living habits, such as excessive drinking and smoking, which are lifestyle risk factors for osteoporosis [21, 22]. Besides, men also tend to have higher incidence rate of many chronic diseases that will affect BMD, such chronic obstructive disease (COPD) and diabetes [23]. Due to smoking and occupational exposure, the prevalence of COPD in men is consistently higher than that in women [24]. The prevalence of osteoporosis in COPD patients is significantly higher than that in healthy controls [25]. Also, the limitation of chest diastolic function in patients with osteoporosis will inhibit respiratory function and aggravate COPD [26], which in return decreases BMD. Hypoxic state [27], Vitamin D deficiency [28], and lack of activity are the main reasons to induce bone loss in patients with chronic COPD. Avoid related lifestyle risk factors for these chronic diseases and for osteoporosis will help to reduce the related burden in men.

Clinically, osteoporosis in men have been overlooked for too long [29]. More attention has been paid to the prevention and treatment of osteoporosis in postmenopausal women, while ignoring the disease in men [30]. For osteoporosis in women, menopause is a clear time point that inform clinicians to pay increased attention to BMD in women. When indicated, anti-osteoporotic therapies can be initiated to avoid bone loss. However, there are few studies or guidelines to help clinicians to determine when to perform osteoporosis screening in men, and when to initiate the treatment of osteoporosis to prevent osteoporotic fractures [7], which are associated with substantial disabilities and socioeconomic burden [2]. Existing guidelines suggest that men over 70 years of age should have dual-energy X-ray absorptiometry measurement, which is the gold standard for determining BMD and can be used to evaluate the efficacy of drug intervention [31, 32]. For men aged 50 to 69, BMD should be examined only if they have one or more of the following risk factors: disease history such as hypogonadism, delayed puberty, hyperthyroidism, hyperparathyroidism, rheumatoid arthritis or COPD; Drug use such as glucocorticoids or gonadotropin-releasing hormone agonist; and lifestyle risk factors such as excess alcohol drinking or smoking [33]. However, as the burden of osteoporosis is heavier in men, it remains to determine whether the screening of BMD in elderly men without risk factors should be advanced in old age to reduce the burden. The balance between health benefits and economic cost of early screening should also be taken into consideration in future studies.

This study has some limitations. Due to the well-established medical facilities and increased medical input, people in high-SDI countries are more likely to have regular screening for BMD, which helps to detect more osteoporosis cases, especially those with latent fractures. In low-income countries, patients tend to seek medical care and have BMD tested only after they experience adverse symptoms (back pain, herniated disc, difficulty walking). This results in potential bias when estimating the prevalence and burden of LBMD. In addition, GBD 2019 covers almost all the countries and territories in the world, and the determination of LBMD may be affected by the diagnostic standards, diagnostic equipment, and physician’s awareness of BMD screening; Thirdly, death certificate is used as one of the data sources. Although it is an important source of public health data, it may be misclassified because it is difficult to determine the potential cause of death [34].

## Conclusion

LBMD and osteoporosis in men has been overlooked for long. Men have lower prevalence of LBMD than women, but the mortality and disability rates are significantly higher than that in women. The higher burden of osteoporosis in men can be explained by high-energy work and activities, more lifestyle risk factors, earlier decline in BMD during aging, and clinical overlook of such disease in men. More attention should be paid to osteoporosis in men, and guidelines based on high-level evidence are in need to guide the screening, prevention and management of osteoporosis in men.

## Supplementary Information

**Additional file 1.** 

**Additional file 2.** 

**Additional file 3.** 

**Additional file 4.** 

**Additional file 5:** **Supplementary Table 1.** Specific SDI values in 204 countries.

**Additional file 6:** **Supplementary Table 2.** Specific SEV values and differences between men and women in 204 countries.

**Additional file 7:** **Supplementary Table 3.** Specific ASDR values and differences between men and women in 204 countries.

**Additional file 8:** **Supplementary Table 4.** Specific ASMR values and differences between men and women in 204 countries.

## Data Availability

The datasets generated and analyzed during the current study are available in the publicly accessible database Global Burden of Disease Study 2019 (GBD 2019). The specific link is https://vizhub.healthdata.org/gbd-results/. "Risk factor" or "Summary exposure value (SEV)" can be selected for the "GBD Estimate" plate. "Deaths" and "DALYs" can be selected for the "Measure" plate. "Number" and "Rate" can be selected for the "Metric" plate. "Low bone mineral density" can be selected for the "Risk" plate. The "Cause" plate can select various causes of injuries, such as "Transport injuries", "Unintentional injuries" and "Self-harm and interpersonal violence". The "Location" plate can select options according to income, SDI, region, etc. "All ages", "Age- standardized" and different age groups can be selected for the "age". There are also options for "Sex" and "Year".
