# Supplementary figures and images for: Differences in the global exposure, mortality and disability of low bone mineral density between men and women: the underestimated burden in men
Source: BMC Public Health. 2023 May 29;23:991. doi: 10.1186/s12889-023-15947-7 (PMC10226255; doi:10.1186/s12889-023-15947-7)

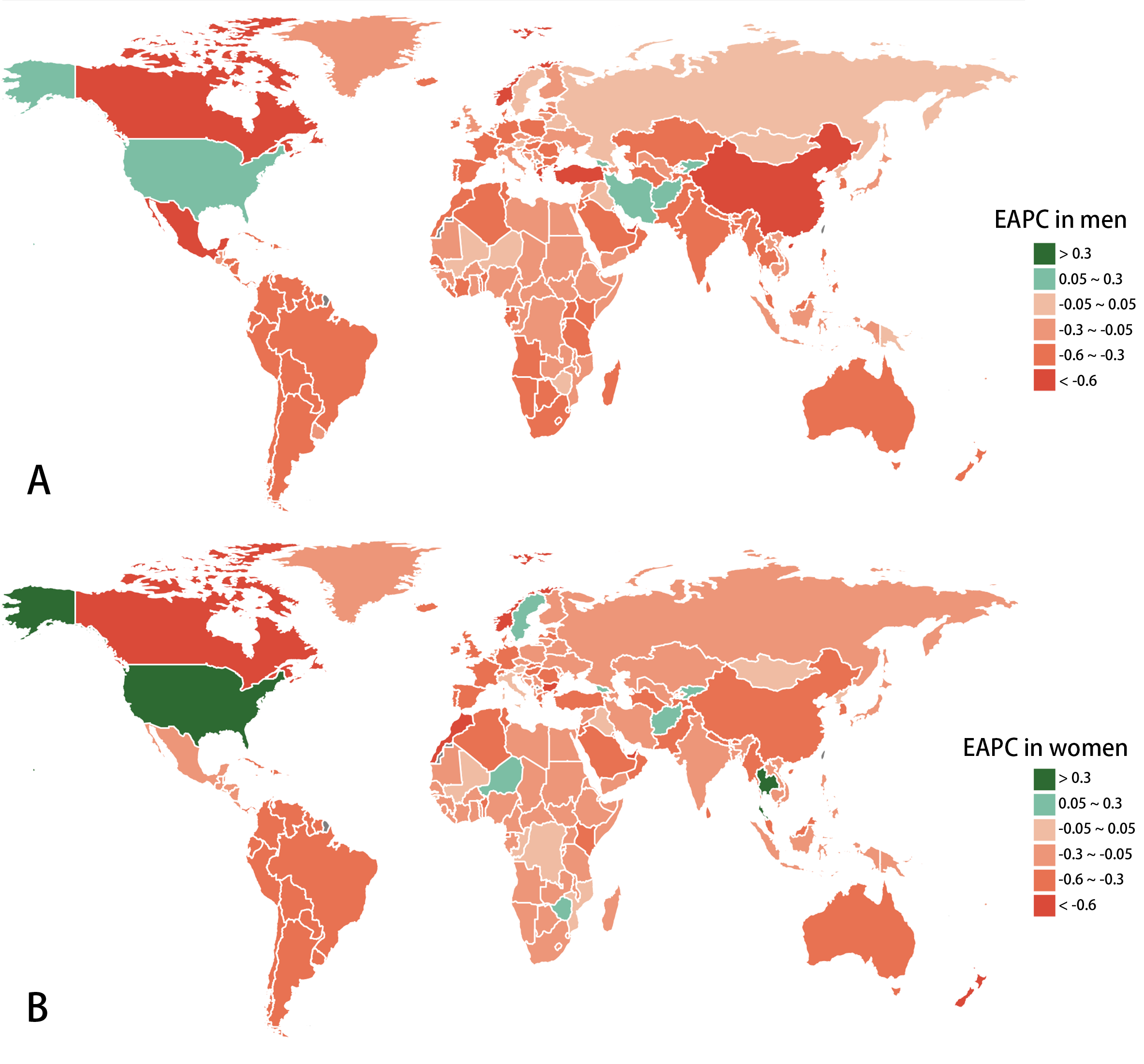

Supplement: Supplementary file 1 — Additional file 1. [file 12889_2023_15947_MOESM1_ESM.tif]

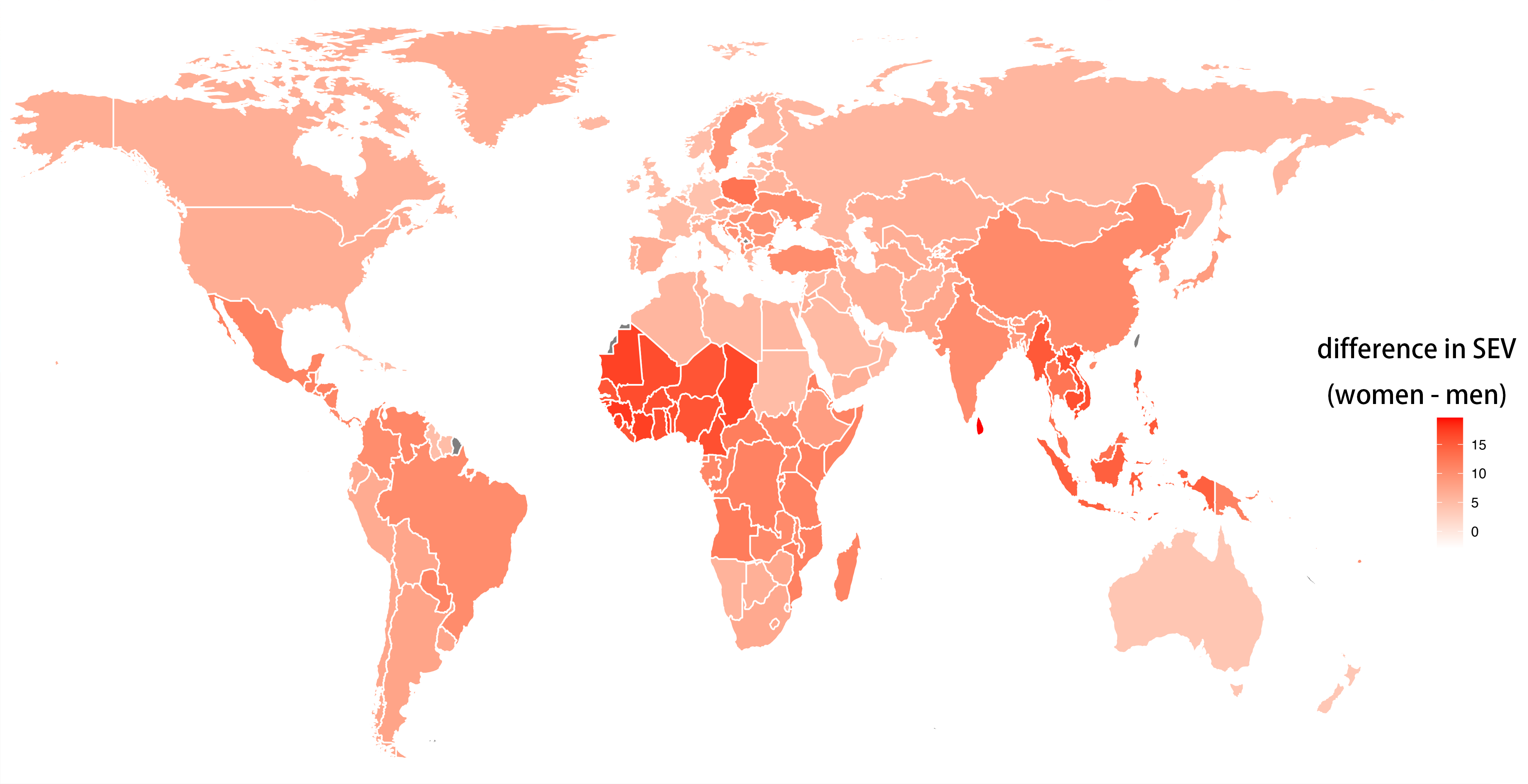

Supplement: Supplementary file 2 — Additional file 2. [file 12889_2023_15947_MOESM2_ESM.tif]

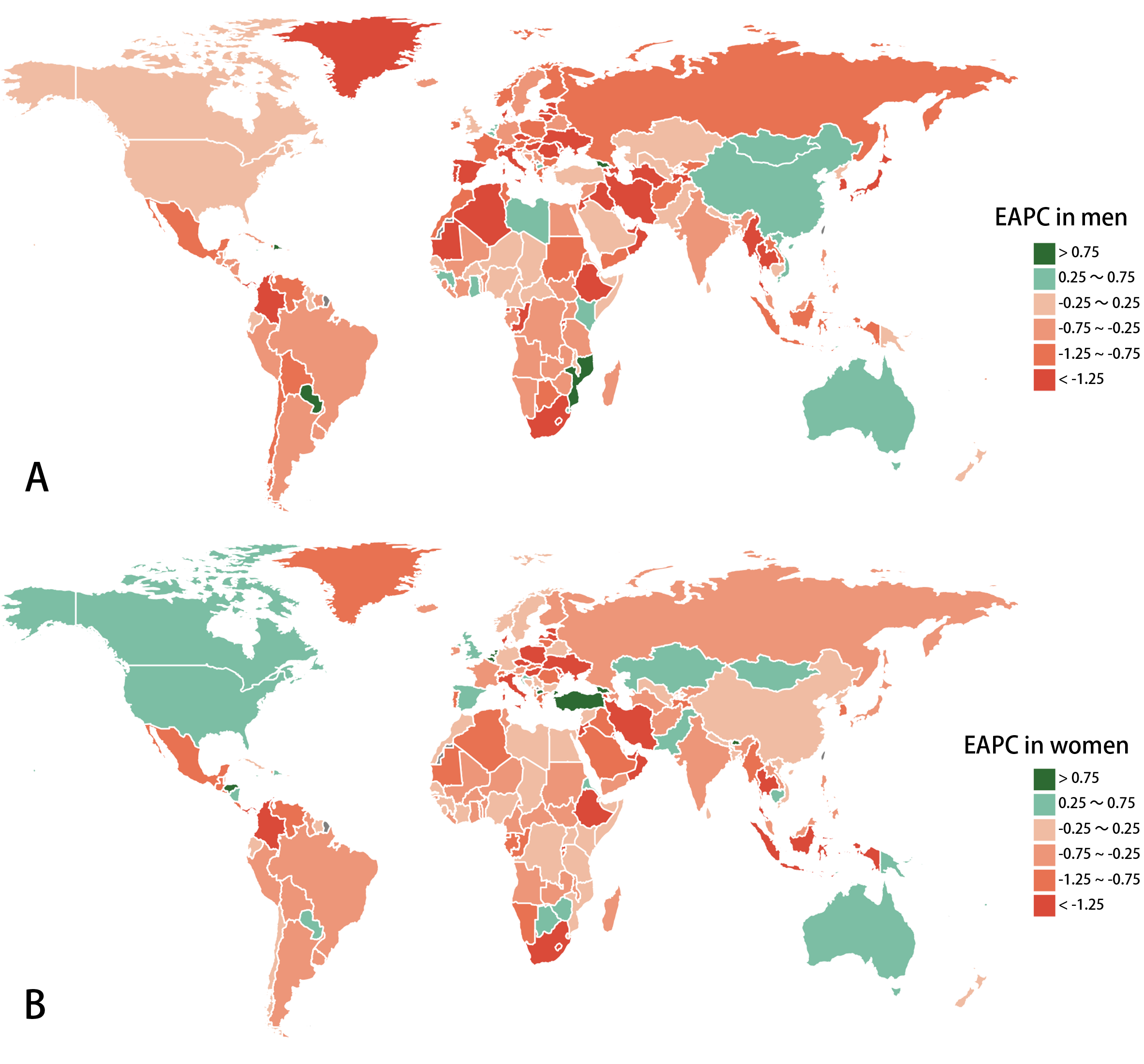

Supplement: Supplementary file 3 — Additional file 3. [file 12889_2023_15947_MOESM3_ESM.tif]

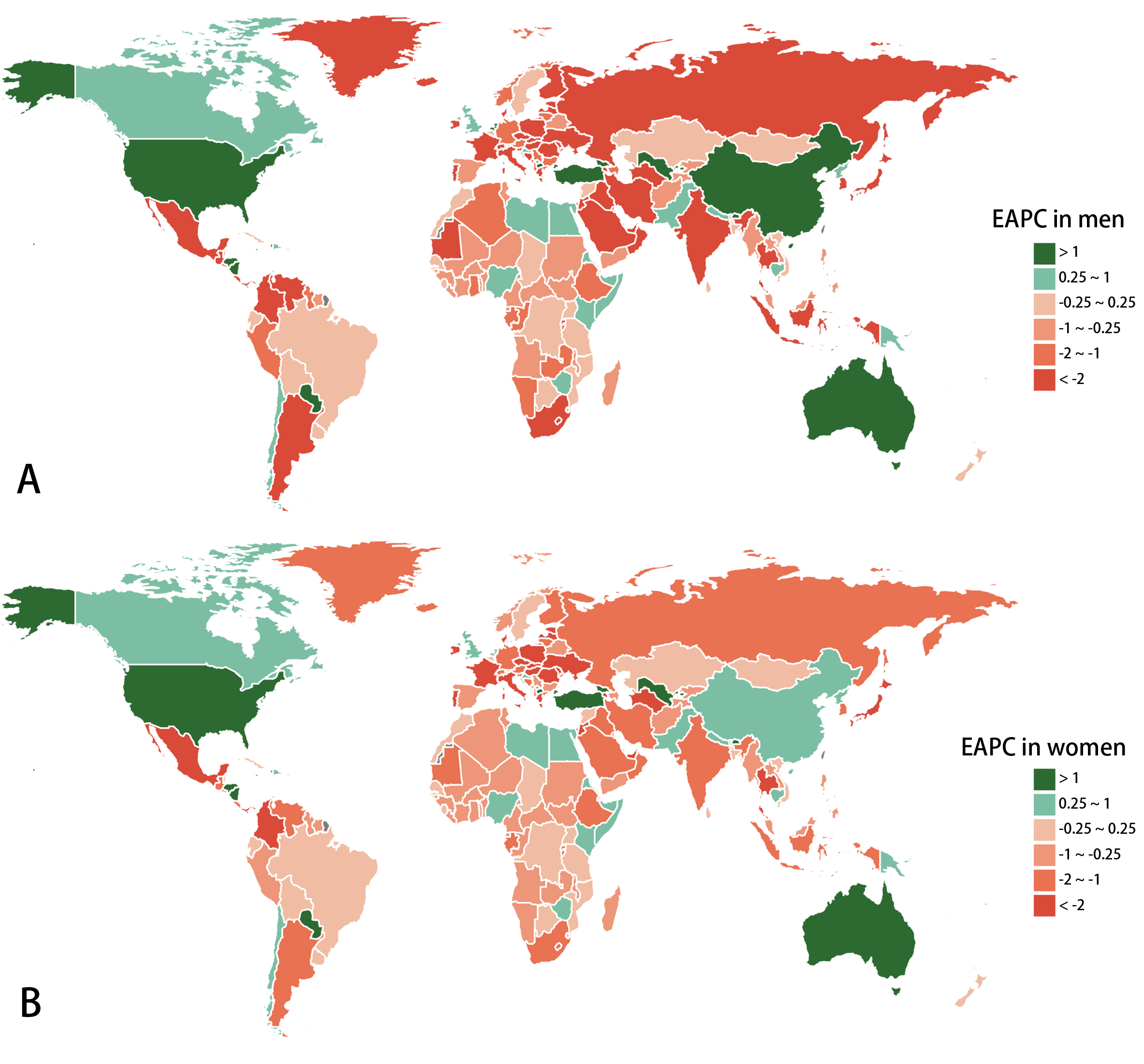

Supplement: Supplementary file 4 — Additional file 4. [file 12889_2023_15947_MOESM4_ESM.tif]
